# Association between rural-to-urban migration and the cognitive aging trajectories of older Chinese adults: results from a prospective cohort analysis

**DOI:** 10.1186/s12877-020-01772-9

**Published:** 2020-09-21

**Authors:** Jinzhao Xie, Jing Liao, Jing Zhang, Jing Gu

**Affiliations:** 1 Department of Medical Statistics, School of Public Health, Sun Yat-sen University, 2nd Zhongshan Road, Guangzhou, Guangdong China; 2 Sun Yat-sen Global Health Institute, School of Public Health and Institute of State Governance, Sun Yat-sen University, 2nd Zhongshan Road, Guangzhou, Guangdong China

**Keywords:** Internal migration, Older adults, Cognitive aging, Cohort analysis

## Abstract

**Background:**

Increasingly, older Chinese adults from rural areas are moving to urban areas to live with their children who have already migrated to these areas. However, few studies have examined this pattern of migration and its effects on cognitive function. We aimed to investigate the association between domestic rural-to-urban migration and the trajectories of cognitive function in older Chinese adults, as well as the factors contributing to these association.

**Methods:**

Data for this study were drawn from three waves of the China Health and Retirement Longitudinal Study. Migrants were defined as participants who had rural *hukou* status (under China’s household registration system) but resided in an urban area. Cognitive functions were measured using an adapted Chinese version of the Mini-Mental State Examination. We used multilevel linear regression models to examine the association between internal migration and cognitive function trajectories.

**Results:**

The study included 3876 Chinese adults aged ≥60 years at baseline. Compared with their rural non-migrant counterparts, migrants (*n* = 850) had higher levels of education and reported more interactions with family. Additionally, female migrants were more likely to participate in leisure activities. All cognitive function scores declined over time, but no significant differences were observed in rates of cognitive decline between migrants and non-migrants, regardless of sex. Female migrants exhibited significantly better performance in terms of total cognition (*β* = 0.77, *P* < .001) and mental status (*β* = 0.68, *P* < .001) than female non-migrants, whereas no inter-group difference was observed regarding memory (*β* = 0.09, *P* > .05). Among the male subjects, no significant differences in cognitive function levels were observed between migrants and non-migrants. A series of adjusted models revealed that psychosocial factors such as residing with children, caring for grandchildren, depression and participation in leisure activities partly explained the association between migration and cognition in women.

**Conclusions:**

Rural-to-urban migration was positively associated with cognitive functions only in women. However, this pattern did not affect the rate of cognitive decline in either sex. Our findings provide directions for tailored interventions improving cognitive functions of older adults and rural non-migrating older adults, especially female non-migrants.

## Background

Estimates indicate that more than 10.4 million individuals with cognitive impairment resided in China in 2016 [1]. Cognitive impairment is associated with a poor quality of life among older adults and imposes heavy burdens on families and societies [2]. In 2018, the number of Chinese adults aged ≥60 years was 249 million, and this group accounted for 17.9% of the total population [3]. Potentially, Chinese health and social care systems may be unprepared to meet the increasing needs of older patients with cognitive impairment. Moreover, the unstable ‘4–2-1’ family structure (four grandparents, two parents, and one child) and the substantial number of working-age adults who migrate from rural to urban areas for employment have undermined traditional practices of family care [4]. Increasingly, older Chinese adults are moving to live near their migrant children, and many provide care for their grandchildren [5]. This unique trend in migration is associated with significant changes in the living environments and social networks of older Chinese adults, which may have important but not fully understood effects on their health and quality of life [6, 7].

The extent to which migration may affect cognitive aging remains unclear [8]. Various aspects may contribute to the effects of migration on the cognition of older adults, including socioeconomic status, physical and mental health and behavioural and environmental factors [9]. Older adults who migrate to cities alongside their adult children may benefit from increased family interactions, support from family members and the opportunity to provide intergenerational care to their grandchildren [9]. Moreover, urban environments provide more opportunities for various leisure activities, which may also contribute to the maintenance of cognitive function [10]. However, some empirical studies have also found that rural-to-urban elderly migrants adapted poorly to aspects of their daily lives [7] and were likely to lose their original social networks, which increased their risk of depression [11]. The association between migration and cognitive function change may show sex-related differences, as women tend to be more involved in family and social activities than men [12, 13]. Consequently, women may be more likely to experience the cognitive benefits associated with these forms of engagement [14–16].

Most previous studies have explored the relationship between international migration and changes in cognitive function; very few have focused on internal migration [8]. In the US, a retrospective cohort study of 1789 Hispanic-American participants indicated no association between migration and cognitive function [17]. Another study of 1085 participants in Europe observed poorer cognitive function in non-European migrants than in local citizens [18]. In China, a 12-year longitudinal study indicated that rural-to-urban and rural residents exhibited more rapid declines in cognitive function than urban residents [19]. In that study, rural-to-urban migrants were compared with native urban citizens rather than with their rural non-migrant counterparts, although the latter group might be more comparable. Moreover, the effects of psychosocial factors on the association between migration and cognitive function were not explored.

Despite the increasing internal rural-to-urban migration of older adults in China, evidence regarding the potential association between migration and cognition remains lacking. Additionally, longitudinal studies are needed to expand the body of evidence regarding the long-term effects of migration on cognitive aging trajectories. This study aimed to investigate the sex-specific association between internal rural-to-urban migration and cognitive aging trajectories and to explore the potential contributing psychosocial factors in a nationally representative longitudinal sample of community-dwelling older Chinese adults, which refer to those aged ≥60 years. We hypothesised that rural-to-urban elderly migrants would exhibit better cognitive function and a slower rate of cognitive decline than their non-migrant rural counterparts. Furthermore, we hypothesised that these associations would be more evident in women than in men and that this difference would be partly attributable to increased family interactions and social engagements.

## Methods

### Study sample

This study used data from three waves of the China Health and Retirement Longitudinal Study (CHARLS 2011–2015), whose design was based on the Health and Retirement Study (HRS) in the US. The CHARLS comprised a nationally representative sample of adults in China aged ≥45 years. The CHARLS sample was obtained using four-stage stratified sampling with the probability-proportional-to-size (PPS) technique [20]. The baseline survey covered 28 provinces, 150 counties/districts and 17,708 respondents from 10,257 households and was conducted between June 2011 and March 2012. Two follow-up interviews were conducted in 2013 and 2015. We restricted our sample to 3876 respondents who met the following criteria: (1) aged ≥60 years at baseline, (2) completion of all three study waves (2011–2015), (3) no history of diseases with potentially strong effects on cognitive function (e.g., cancer, stroke, memory-related disease) at baseline and (4) rural *hukou* status at baseline.

### Measures

#### Migrants and non-migrants

We divided our sample into two groups, migrants and non-migrants, based on the *hukou* system, which was used to classify rural and urban residents in previous studies [21, 22]. Older adults (≥60 years) with rural *hukou* status who resided in urban areas during all three study waves (*n* = 850) were defined as rural-to-urban elderly migrants. Non-migrants were defined as respondents with rural *hukou* status who resided in rural areas during the three study waves (*n* = 3026).

#### Cognitive function

Cognitive function was measured using an adapted Chinese version of the Mini-Mental Status Examination (MMSE), which included similar concepts to those used to measure cognitive function in the US Health and Retirement Study (HRS) [23]. According to previous publications [24–26], we divided cognitive function into two dimensions: episodic memory and mental status. We generated an episodic memory score (range: 0–10) as the average of the immediate and delayed recall scores. The mental status score (range: 0–11) was based on the following three items: figure drawing, serial subtraction of 7 from 100 (up to five times) and the ability to identify the date (month, day, year), day of the week and season of the year. The total cognition score, which incorporated both dimensions, ranged from 0 to 21. A higher score indicated better cognitive function.

#### Psychosocial factors

Data on the psychosocial factors were obtained from the baseline survey. The psychosocial factors comprised the *family connections, social attachment* and *depression*. *Family connections* was determined by whether the respondent was coupled, lived with his/her adult children and had provided any care to his/her grandchildren. According to the CHARLS code book, if the respondent reported that he/she co-resided with any adult child, regardless of whether he/she took care of grandchildren, the respondent was classified as ‘lives with children’. Caring for grandchildren was defined as the provision of care to any grandchildren younger than 16 years during the past year, regardless whether the respondent lived with grandchildren.

The measure of *social attachment* was adapted from the definition provided in the English Longitudinal Study of Aging (ELSA). Specifically, that study divided social attachment into four domains: civic participation, leisure activities, cultural engagement and social networks. To accommodate the Chinese social background of our subjects, we excluded cultural engagement, which was assessed by the frequency with which the participants reported visiting art galleries, museums or exhibitions and attending theatres, concerts, operas and cinemas, from our analysis. Civic participation was defined as the participation in activities associated with a community-related organisation or in volunteer or charity activities. Subjects who reported that they had participated in one of the above-mentioned activities within 1 month before the interview were classified as having civic participation. Participation in leisure activities was defined as playing mah-jong, cards or chess; visiting a community; attending an athletic, social or other type of club; or attending an educational or training course within 1 month before the interview. The domain of social network was restricted to friendships and was defined as interactions with friends within 1 month before the interview. Other core social networks experienced by elders were measured under the domain of *family connections*.

*Depression* was measured using the 10-item Centre for Epidemiologic Studies Depression Scale (CES-D-10). The total scores ranged from 0 to 30, and a higher score indicated more severe depression.

#### Covariates

The subjects’ demographic characteristics, socioeconomic and health statuses and health behaviours were considered as covariates in our study. In accordance with prior CHARLS studies and the distribution of educational attainment among older Chinese adults, the subjects were classified into four educational levels: illiterate; some primary school (not completed); finished primary school; and higher than primary school [27, 28]. Household income was defined as the sum of all annual income at the household level and was stratified into three levels (low, medium and high) according to the lower and upper quartiles. Retirement status was dichotomised as retired or not retired. Retirement was defined as a history of employment (including agricultural and non-agricultural work) and a current status of no longer working, while non-retirement was defined as participation in current employment (agricultural and non-agricultural) or no history of such work throughout one’s lifetime.

Health status was assessed according to the number of activities of daily living (ADL) in which the subject experienced disability and chronic disease status. ADLs were determined as the number of activities during which the subject experienced difficulties (range: 0–6). Chronic disease status was determined from self-reported diagnoses. According to previous studies, smoking, alcohol consumption and afternoon napping may affect cognitive function in the elderly [9, 23, 29]. Therefore, we considered these three items as health behaviours. The subjects were categorised as non-smokers, light/moderate smokers (< 20 cigarettes per day currently or a history of smoking) or heavy smokers (≥20 cigarettes per day currently). They were further categorised into three alcohol consumption categories: non-drinkers, ≤1 drink per month or > 1 drink per month. The subjects were further categorised as non-nappers, short nappers (< 30 min), moderate nappers (30–90 min) or extended nappers (> 90 min) [29].

### Statistical methods

The characteristics of the sample were described according to sex and migrant status. Continuous variables are reported as means and standard deviations, while categorical variables are reported as percentages. The *t*-test was used to compare normally distributed continuous variables. The chi-square test was used to compare the nominal variables, namely retirement, family connections, social attachment and chronic disease, while the rank-sum test was used to compare the ordinal variables of age group, education level, household annual income, smoking, alcohol consumption and afternoon napping.

We examined differences in the subjects’ cognitive function trajectories using multilevel linear regression analyses in which the follow-up wave was set as the first level (low level) and coded as 0, 1 or 2 to represent the longitudinal term, and individuals were set as the second level (high level). We assumed that individuals would have different baseline levels of cognitive function and different rates of cognitive decline. Therefore, we estimated the random coefficient models. First, we detected the difference in cognitive trajectories between migrants and non-migrants and evaluated the presence of a sex-specific difference by establishing a model that included the interacting terms of time, migration status and sex. Because we identified a significant interaction between sex and the migration status with respect to the total cognition and mental status scores (see Additional file 1: Table S1), we stratified all of the analyses by sex.

Given the observed difference in cognitive function between female migrants and non-migrants, we constructed a series of adjustment models to explore the possible underlying factors. In these adjustment models, the psychosocial factors and covariates were entered at level 2 (interpersonal level). Model 1 was adjusted for the age group and time of follow-up. Model 2 comprised model 1 plus the socioeconomic status, while models 3 and 4 added psychosocial factors. Finally, model 5 included the health status and health behaviours. We used multiple imputation by chained equations (MICE) to impute any missing values. This process was performed using R version 3.4.5 with the ‘mice’ package. We also conducted a sensitivity analysis by running models in which the missing values had not been imputed, and achieved similar results. In all of the analyses, statistical significance was based on a two-tailed *P* value < 0.05. All of the analyses were performed using R software version 3.4.5.

## Results

### Baseline characteristics of the overall sample and subgroups stratified by migration status and sex

The total sample comprised 3876 participants (52.2% female, 21.9% migrants), with an average baseline age of 67.5 ± 6.5 years. Of the participants, 43.7% were illiterate, 30.3% had retired, 78.1% lived in a coupled household, 48.7% lived with children and 39.9% had cared for grandchildren in the past year. Few reported civic participation (0.9%), 14.7% had participated in leisure activities during the past month and 32.7% reported interaction with friends during the previous month. Nearly three quarters of the participants had chronic diseases, and the average depression score was 9.1 ± 6.5. More than half of the participants were non-smokers and non-drinkers, and nearly half did not take naps. The average total cognition score was 9.2 ± 3.8.

Among the female subjects, migrants accounted for 23.6% (*n* = 478) of the participants. Female migrants were more likely to live with children (58.6% vs 47.5%, *P* < 0.001), care for grandchildren (48.1% vs 34.5%, *P* < 0.001) and participate in leisure activities (16.3% vs 8.6%, *P* < 0.001) than were female non-migrants. Among the male subjects, migrants accounted for 20.1% (*n* = 372) of the participants and were more likely than male non-migrants to live with children (57.8% vs 44.5%, *P* < 0.001) and care for grandchildren (52.2% vs 39.7%, *P* < 0.001). However, there was no significant difference in the frequency of participation in leisure activities between male migrants and non-migrants (20.4% vs 19.1%, *P* = 0.598).

Although female migrants had better baseline total cognition (8.8 ± 3.8 vs 8.1 ± 3.8, *P* = 0.001) and mental status scores (5.8 ± 3.0 vs 5.2 ± 3.0, *P* < 0.001) than female non-migrants, similar differences were not observed among the male participants. Moreover, no significant inter-group differences in episodic memory were observed. The detailed baseline characteristics are presented in Table 1.

**Table 1:** Baseline Characteristics of the Overall Sample and According to Migration Status by Sex

Variables	Total (*n* = 3876)	Female (*n* = 2024)		*P*	Male (*n* = 1852)		*P*
		Non-migrant (*n* = 1546)	Migrant (*n* = 478)		Non-migrant (*n* = 1480)	Migrant (*n* = 372)	
**Demographic**							
Age, mean ± SD	67.5 ± 6.5	67.6 ± 6.6	68.2 ± 7.2	0.061	67.3 ± 6.2	67.1 ± 6.0	0.556
Age group, *n* (%)				0.193			0.693
< 65	1622 (41.8)	635 (41.1)	191 (40.0)		629 (42.5)	167 (44.9)	
65 ~ 70	1190 (30.7)	492 (31.8)	138 (28.9)		450 (30.4)	110 (29.6)	
> 70	1064 (27.5)	419 (27.1)	149 (31.2)		401 (27.1)	95 (25.5)	
Education level, n (%)				0.032			0.001
Illiterate	1694 (43.7)	986 (63.8)	270 (56.5)		376 (25.4)	62 (16.7)	
Some primary school	860 (22.2)	280 (18.1)	100 (20.9)		362 (24.5)	118 (31.7)	
Finished primary school	943 (24.3)	222 (14.4)	83 (17.4)		504 (34.1)	134 (36.0)	
Higher than primary school	379 (9.8)	58 (3.8)	25 (5.2)		238 (16.1)	58 (15.6)	
Retired, n (%)	1173 (30.3)	544 (35.2)	213 (44.6)	<.001	288 (19.5)	128 (34.4)	<.001
Household annual income, n (%)				<.001			<.001
Low	1098 (28.3)	473 (30.6)	125 (26.2)		429 (29.0)	71 (19.1)	
Medium	2074 (53.5)	821 (53.1)	241 (50.4)		810 (54.7)	202 (54.3)	
High	704 (18.2)	252 (16.3)	112 (23.4)		241 (16.3)	99 (26.6)	
**Family connections**, *n* (%)							
Coupled household	3026 (78.1)	1128 (73.0)	327 (68.4)	0.061	1238 (83.6)	333 (89.5)	0.006
Living with children	1889 (48.7)	735 (47.5)	280 (58.6)	<.001	659 (44.5)	215 (57.8)	<.001
Caring for grandchildren	1545 (39.9)	534 (34.5)	230 (48.1)	<.001	587 (39.7)	194 (52.2)	<.001
**Social attachment**, *n* (%)							
Civic participation	33 (0.9)	11 (0.7)	4 (0.8)	0.999	14 (0.9)	4 (1.1)	0.999
Leisure activities	569 (14.7)	133 (8.6)	78 (16.3)	<.001	282 (19.1)	76 (20.4)	0.598
Friendships	1269 (32.7)	554 (35.8)	161 (33.7)	0.420	452 (30.5)	102 (27.4)	0.266
**Depression**, (mean ± SD, 0–30)	9.1 ± 6.5	10.3 ± 6.8	9.0 ± 6.7	<.001	8.2 ± 6.0	7.5 ± 5.7	0.039
**Health**							
ADLs, (mean ± SD, 0–6)	0.5 ± 1.1	0.6 ± 1.3	0.5 ± 1.1	0.035	0.4 ± 1.0	0.3 ± 0.7	0.069
Chronic diseases	2757 (71.1)	1144 (74.0)	348 (72.8)	0.646	1018 (68.8)	247 (66.4)	0.411
**Health behaviours**							
Smoking, *n* (%)				0.115			0.477
Non-smokers	2582 (66.6)	1457 (94.2)	438 (91.6)		553 (37.4)	134 (36.0)	
Light/moderate smokers	601 (15.5)	64 (4.1)	30 (6.3)		411 (27.8)	96 (25.8)	
Heavy smokers	693 (17.9)	25 (1.6)	10 (2.1)		516 (34.9)	142 (38.2)	
Alcohol consumption, *n* (%)				0.954			0.183
Non-drinker	2665 (68.8)	1349 (87.3)	418 (87.4)		713 (48.2)	185 (49.7)	
≤ 1 drink per month	239 (6.2)	65 (4.2)	21 (4.4)		115 (7.8)	38 (10.2)	
> 1 drink per month	972 (25.1)	132 (8.5)	39 (8.2)		652 (44.1)	149 (40.1)	
Afternoon napping, *n* (%)				0.066			0.507
Non-napper	1878 (48.5)	876 (56.7)	240 (50.2)		602 (40.7)	160 (43.0)	
Short napper	327 (8.4)	114 (7.4)	35 (7.3)		149 (10.1)	29 (7.8)	
Moderate napper	1111 (28.7)	386 (25.0)	145 (30.3)		467 (31.6)	113 (30.4)	
Extended napper	560 (14.4)	170 (11.0)	58 (12.1)		262 (17.7)	70 (18.8)	
**Cognition**, mean ± SD							
Total cognition (range 0–21)	9.2 ± 3.8	8.1 ± 3.8	8.8 ± 3.8	0.001	10.3 ± 3.7	10.5 ± 3.4	0.274
Mental status (range 0–11)	6.2 ± 3.1	5.2 ± 3.0	5.8 ± 3.0	<.001	7.2 ± 2.9	7.5 ± 2.7	0.086
Episodic memory (range 0–10)	3.0 ± 1.7	2.9 ± 1.7	3.0 ± 1.7	0.651	3.1 ± 1.6	3.0 ± 1.6	0.556

*Abbreviations*: *SD* standard deviation, *SES* socioeconomic status, *ADL* activity of daily living disability

### Sex-specific differences in cognitive trajectories between migrants and non-migrants

**Fig. 1:**
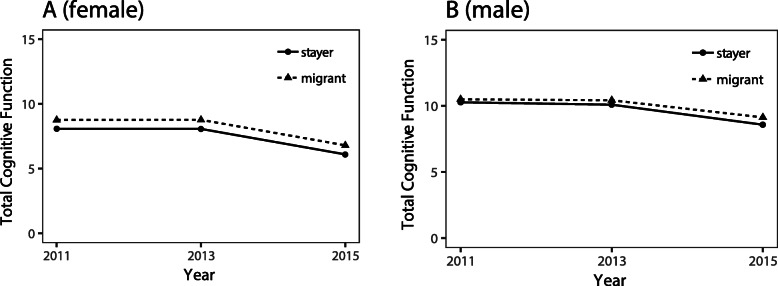
The sex-specific differences in total cognitive function trajectories function by migrant status. **a** Relationship between time (horizontal axis) and total cognitive function (range:0–21; vertical axis) of female according to migrant status. The dotted line and solid line represent migrant and stayer respectively, the dots represent the mean total cognitive function of each fellow-up point. **b** Association between time and total cognitive function of male according to migrant status. Over all the total cognitive function of male was higher than that of female. The cognitive performance of migrants was better than stayers, which was more pronounced in female

Table 2 presents the results of the multilevel model analyses. After the analyses were adjusted for age groups, the time terms in all models were significantly negative, indicating that all cognitive functions declined with time. Among women, migrants achieved better scores for total cognition (*β* = 0.77, *P* < 0.001) and mental status domains (*β* = 0.68, *P* < 0.001) except episodic memory (*β* = 0.09, *P* > 0.05) when compared with non-migrants. Among men, however, there were no significant differences in any of the cognitive function levels between migrants and non-migrants. The interacting terms of migration and time were not significant in all models, indicating that the differences in the rates of cognitive function decline between migrants and non-migrants were not significant. The sex-specific differences in total cognitive function according to migrant status are illustrated in Fig. 1.

**Table 2:** Sex-specific Differences in Cognitive Trajectories Between Migrants and Non-migrants

Mixed Effect	β (SE)		
	Total cognition	Mental status	Episode Memory
**Female**			
Fixed effect			
Constant	9.40 (0.12)^***^	6.09 (0.10)^***^	3.34 (0.05)^***^
Time	−0.99 (0.05)^***^	− 0.59 (0.05)^***^	− 0.40 (0.03)^***^
Migrant	0.77 (0.18)^***^	0.68 (0.15)^***^	0.09 (0.08)
Age65 ~ 70 (Ref. Age60~)	−0.83 (0.16)^***^	− 0.47 (0.12)^***^	− 0.39 (0.06)^***^
Age > 70 (Ref. Age60~)	−2.68 (0.17)^***^	−1.69 (0.13)^***^	−1.10 (0.07)^***^
Migrant x Time	−0.01 (0.11)	− 0.03 (0.09)	0.03 (0.05)
Random effect			
ID	2.27	1.72	0.97
Time	0.26	0.12	0.35
**Male**			
Fixed effect			
Constant	11.39 (0.12)^***^	7.88 (0.09)^***^	3.54 (0.05)^***^
Time	−0.85 (0.05)^***^	−0.49 (0.04)^***^	−0.36 (0.03)^***^
Migrant	0.16 (0.19)	0.25 (0.15)	−0.09 (0.09)
Age65 ~ 70 (Ref. Age60~)	−0.68 (0.16)^***^	−0.36 (0.12)^**^	− 0.35 (0.07)^***^
Age > 70 (Ref. Age60~)	−2.52 (0.17)^***^	−1.61 (0.13)^***^	−1.00 (0.07)^***^
Migrant x Time	0.16 (0.12)	0.06 (0.10)	0.11 (0.06)
Random effect			
ID	2.03	1.53	0.83
Time	0.45	0.31	0.27

^*^ *P* < 0.05; ^**^ *P* < 0.01; ^***^ *P* < 0.001;

### Sex-specific contributions of psychosocial factors to the association between migration and cognitive function

A series of adjustment models revealed the contributions of psychosocial factors to differences in the cognitive function levels between migrants and non-migrants. Regarding total cognition in the female participants, after controlling for the factors included in models 1–5 in a stepwise manner, the estimated effect of migration remained positive (model 1: *β* = 0.78, *P* < 0.001; model 2: *β* = 0.47, *P* < 0.001; model 3: *β* = 0.48, *P* < 0.001; model 4: *β* = 0.35, *P* < 0.01; model 5: *β* = 0.32, *P* < 0.05). The results of model 4 revealed that living with children (*β* = − 0.28, *P* < 0.05) and depression (*β* = − 0.06, *P* < 0.001) were associated with a lower total cognition level, while participation in leisure activities (*β* = 0.87, *P* < 0.001) was positively associated with the cognitive function level. In model 4, the estimated effect of migration remained positive, although the decrease in this value from 0.47 in model 2 to 0.35 in model 4 indicated that psychosocial factors explained 25.5% of the difference in the total cognitive function level between migrants and non-migrants. After all of the covariates were adjusted in model 5, the estimated effects of living with children, participation in leisure activities and depression remained significant (Table 3). Among the male participants, migration status remained a non-significant factor after controlling for psychosocial factors included in models 2–5. Greater participation in leisure activities and more interactions with friends were associated with a higher total cognitive level (see Additional file 1: Table S3).

**Table 3:** Association Between the Total Cognitive Trajectory and Migration Status in Female Subjects

Mixed Effect	β (SE)				
	Model 1	Model 2	Model 3	Model 4	Model 5
Fixed effect					
constant	9.39 (0.12)^***^	8.08 (0.16)^***^	8.00 (0.20)^***^	8.55 (0.23)^***^	8.49 (0.25)^***^
Time	−0.99 (0.05)^***^	−0.99 (0.05)^***^	− 0.99 (0.05)^***^	−0.99 (0.05)^***^	− 0.99 (0.05)^***^
Migrant (Ref. Non-migrants)	0.78 (0.16)^***^	0.47 (0.14)^***^	0.48 (0.14)^***^	0.35 (0.14)^**^	0.32 (0.14)^*^
Age65 ~ 70 (Ref. Age60~)	−0.83 (0.16)^***^	−0.85 (0.14)^***^	− 0.85 (0.14)^***^	−0.79 (0.14)^***^	− 0.78 (0.14)^***^
Age > 70 (Ref. Age60~)	−2.68 (0.17)^***^	−1.72 (0.16)^***^	−1.66 (0.16)^***^	−1.65 (0.16)^***^	− 1.61 (0.16)^***^
**SES**					
Education level (Ref. illiterate)					
Some primary school		2.09 (0.15)^***^	2.09 (0.15)^***^	2.06 (0.15)^***^	2.03 (0.15)^***^
Finished primary school		3.88 (0.17)^***^	3.88 (0.17)^***^	3.73 (0.17)^***^	3.69 (0.17)^***^
Higher than primary school		5.17 (0.30)^***^	5.14 (0.30)^***^	4.92 (0.29)^***^	4.88 (0.29)^***^
Retired (Ref. no)		−0.09 (0.13)	−0.05 (0.13)	− 0.06 (0.12)	−0.03 (0.12)
Household annual income (Ref. low)					
Medium		−0.15 (0.13)	−0.10 (0.14)	− 0.21 (0.14)	−0.21 (0.14)
High		0.26 (0.17)	0.41 (0.19)^*^	0.15 (0.19)	0.14 (0.19)
**Family connections**					
Coupled household			0.15 (0.14)	0.16 (0.14)	0.15 (0.14)
Living with children			−0.36 (0.13)^**^	−0.28 (0.12)^*^	−0.29 (0.12)^*^
Caring for grandchildren			0.16 (0.12)	0.19 (0.12)	0.21 (0.12)
**Social attachment**					
Civic participation (Ref.no)				0.59 (0.65)	0.52 (0.65)
Leisure activities (Ref.no)				0.87 (0.19)^***^	0.83 (0.19)^***^
Friendships (Ref.no)				0.25 (0.12)^*^	0.21 (0.12)
**Depression**				−0.06 (0.01)^***^	−0.06 (0.01)^***^
**Health**					
ADLs					−0.12 (0.05)^*^
Chronic diseases (Ref. Non-disease)					−0.05 (0.13)
**Health behaviours**					
Smoking (Ref. Non-smoker)					
Light/moderate smokers					0.30 (0.27)
Heavy smokers					0.44 (0.43)
Alcohol consumption (Ref. Non-drinker)					
≤ 1 drink per month					−0.01 (0.28)
> 1 drink per month					−0.05 (0.21)
Afternoon napping (Ref. Non-napper)					
Short napper					0.65 (0.22)^**^
Moderate napper					0.31 (0.13)^*^
Extended napper					−0.19 (0.18)
Random effect					
ID	2.27	1.76	1.76	1.65	1.64
Time	0.25	0.17	0.16	0.20	0.20
AIC	32,734	32,075	32,069	31,986	31,980

*Abbreviations*: *AIC* Akaike Information Criterion, *SES* socioeconomic status, *ADL* activity of daily living disability

Model 1: Adjusted for age group and time of follow-up; Model 2: Model 1 + socioeconomic status; Model 3: Model 2 + family connections; Model 4: Model3 + social attachment + depression; Model 5: Model 4 + health and health behaviours

^*^ *P* < 0.05; ^**^ *P* < 0.01; ^***^ *P* < 0.001;

Regarding mental status, female migrants had a better mental status than female non-migrants (model 1: *β* = 0.66 *P* < 0.001; model 2: *β* = 0.41, *P* < 0.001; model 3: *β* = 0.41, *P* < 0.001; model 4: *β* = 0.32, *P* < 0.01; model 5: *β* = 0.29, *P* < 0.05; see Additional file 1: Table S2). Participation in leisure activities (model 4: *β* = 0.55, *P* < 0.001) remained significantly associated with a better mental status. Caring for grandchildren (model 4: *β* = 0.21, *P* < 0.05) was also associated with a better mental status in the female participants, whereas living with children (model 4: *β* = − 0.27, *P* < 0.01) and depression (model 4: *β* = − 0.04, *P* < 0.001) were associated with worse mental health. Among the male participants, migration status remained a non-significant factor after controlling for the factors included in models 2–5 in a stepwise manner. Greater participation in leisure activities (model 4: *β* = 0.58, *P* < 0.001) was associated with a higher cognitive level, whereas living with children (model 4: *β* = − 0.25, *P* < 0.05) was associated with a worse mental status (see Additional file 1: Table S4).

## Discussion

We observed a better cognition level among rural-to-urban elderly female (but not male) migrants, compared with their non-migrant counterparts. These differences were evident with respect to the overall level of cognitive function but not the rate of cognitive decline, and were partly explained by variations in the socio-economic status, behavioural and psychosocial factors and other health-related factors. This work indicates the potential directions of specific interventions for internal elderly migrants and identifies the population that requires most attention.

In our study, we did not observe a significant difference in the rate of cognitive decline between migrants and non-migrants, which might be explained by the following reasons. First, as both rural-to-urban migrants and rural non-migrants had rural life experiences, the differences in the rates of cognitive decline between these populations may be less significant than those observed between rural and urban residents [19]. Second, according to Cattell’s categorisation of cognitive abilities and previous studies, fluid abilities such as memory tend to decline linearly from early adulthood and are more difficult to improve or otherwise change in older adults [30–32]. This was consistent with our observations.

We observed a higher cognitive function level in female migrants than in female non-migrants. This might be explained partially by the finding that female migrants were more likely to take care of grandchildren and participate in leisure activities than were their non-migrant counterparts. Moreover, female migrants were less likely to present with depression. According to previous studies, caring for grandchildren and leisure activities were positively associated with cognitive function [10, 14, 15]. However, no significant difference in cognitive function was observed between male migrants and non-migrants, indicating that men and women may not achieve the same benefits from caring for grandchildren. Moreover, our findings show that male migrants were not more likely than non-migrants to participate in leisure activities. Studies of additional reasons for sex-related difference observed in our findings are warranted.

Although both female and male migrants were more likely to provide intergenerational care than non-migrants, our findings suggest that this activity only provided cognitive benefits in terms of the mental status among female migrants. Previous studies have argued that voluntarily providing care to grandchildren might have a positive effect on an elderly caregiver’s cognitive function by enhancing the caregiver’s senses of self-esteem and self-worth and providing a new purpose in later life [33–35]. However, this effect may be somewhat sex-specific and could therefore provide more benefits to female caregivers [14, 15]. Our study yielded similar results. We think that this outcome may be partly attributable to traditional social norms in China, where women are typically expected to be responsible for domestic affairs and family life and to play a nurturing role and serve as kin-keepers, whereas men are expected to fulfil the role of breadwinners. Accordingly, grandfathers who migrate to urban areas specifically to provide care for grandchildren would deviate from this traditional social norm. Therefore, female rural-to-urban elderly migrants would more easily benefit from caring for grandchildren.

Our findings also suggested other explanations regarding the sex-specific difference in cognitive function between migrants and non-migrants. We observed that female elderly migrants performed better in terms of social attachment than female non-migrants, particularly in leisure activities. This finding was inconsistent with those of previous studies, which reported that migrants experienced poor social adaptation and integration [7, 21, 36]. This inconsistency may be related to differences in the selection of the control group, which comprised non-migrant rural elderly people in our study but local citizens in other studies. Compared with rural areas, urban areas feature a wealth of community activities and facilities for leisure activities [37, 38], which provide migrants with more opportunities for participation. Consequently, rural-to-urban migrants have more opportunities for leisure activities. However, increased participation in leisure activities was only observed in female migrants. Compared with men, women tend to have more larger and more varied social networks and to exchange support with a greater number of members in their networks [39]. In contrast, men often depend solely on their spouses and may be less likely to participate in social activities in the community [40]. Our findings showed that living with children was negatively associated with cognitive function. From our data, older adults living with children at baseline reported lower cognitive function (mental status) at both baseline and follow up. Findings that living with children was associated with physical disabilities were reported in other studies [41–43]. This pattern may be attributed to the fact that older adults with physical or cognitive impairment tend to live with their children for support.

Our study had some limitations that should be acknowledged. First, although we used *hukou* status to classify the participants into rural and urban populations in accordance with the Chinese context and previous studies [38, 44, 45], we were unable to obtain detailed information on aspects of migration such as the migration time, process and reasons. Further studies should be conducted to explore the effects of migration based on different reasons on cognitive function. Second, it was difficult to match rural-to-urban migrants with rural non-migrants who shared the same residential location information in the *hukou* system. We believe that given the significant development gap between different regions of China, it would be more reasonable to compare populations that originated in the same region. Third, although this analysis covered a 4-year period, it might not have been long enough to enable the development of differences in the rates of cognitive decline. Fourth, our study explored the associations between baseline psychosocial factors and the subjects’ cognitive trajectory. However, the psychosocial statuses of the subjects may have changed over time. Further studies that explore the effects of time-variant psychosocial factors on cognitive function are warranted. Lastly, our study might have been subject to the ‘healthy migrant effect’. To minimise this effect, however, we adjusted for several health conditions as covariates in our regression models. Still, other potential contributors to the healthy migrant effect may have been overlooked. Further longitudinal studies are warranted to explore the cognitive trajectory of migrants before and after migration and thus control the influence of the healthy migrant effect.

## Conclusions

In conclusion, our research has advanced the body of knowledge regarding the associations between rural-to-urban migration and the cognitive function trajectories of elderly Chinese residents. Moreover, we have revealed a sex-related difference in this association and explored the potential underlying psychosocial factors. Our results indicate that both elderly male migrants and rural non-migrants require more attention and that interventions targeting the preservation of cognitive function in elderly internal migrants should be developed according to these sex-related differences. Finally, we hope that our study will serve as a basis for further studies of the mechanism underlying the relationship between migration and cognitive function in older adults.

## Supplementary information

**Additional file 1: Table S1** Differences in Cognitive Trajectories Between Migrants and Non-migrants. **Table S2** Longitudinal Association Between Mental Status and Migrant for Female. **Table S3** Longitudinal Association Between the Total Cognitive Trajectory and Migrant Status in Male Subjects. **Table S4** Longitudinal Association Between the Mental Status and Migrant Status in Male Subjects.

## Data Availability

The CHARLS datasets, which analysed during the current study, are publicly available at the National School of Development, Peking University (http://charls.pku.edu.cn/en) and can be obtained after submitting a data use agreement to the CHARLS team.

## Abbreviations

ADL: Activities of daily living
CES-D-10: the 10-item Centre for Epidemiologic Studies Depression Scale
CHARLS: China Health and Retirement Longitudinal Study
ELSA: English Longitudinal Study of Aging
HRS: Health and Retirement Study
MMSE: Mini-Mental Status Examination
PPS: Probability-proportional-to-size
