# Supplementary material for: Association between rural-to-urban migration and the cognitive aging trajectories of older Chinese adults: results from a prospective cohort analysis
Source: BMC Geriatr. 2020 Sep 21;20:360. doi: 10.1186/s12877-020-01772-9 (PMC7507287; doi:10.1186/s12877-020-01772-9)
Supplement: Supplementary file 1 — Additional file 1: Table S1 Differences in Cognitive Trajectories Between Migrants and Non-migrants. Table S2 Longitudinal Association Between Mental Status and Migrant for Female. Table S3 Longitudinal Association Between the Total Cognitive Trajectory and Migrant Status in Male Subjects. Table S4 Longitudinal Association Between the Mental Status and Migrant Status in Male Subjects. [file 12877_2020_1772_MOESM1_ESM.docx]

**Table S1** Differences in Cognitive Trajectories Between Migrants and Non-migrants

| Mixed Effect | β (SE) |  |  | |
| --- | --- | --- | --- | --- |
|  | Total cognition | Mental status | Episode Memory | |
| Fixed effect |  |  |  |  |
| Constant | 11.43(0.10)^***^ | 7.91(0.08)^***^ | 3.56(0.05)^***^ |  |
| Time | -0.85(0.05)^***^ | -0.49(0.05)^***^ | -0.36(0.03)^***^ |  |
| Migrant | 0.16(0.22) | 0.25(0.16) | -0.09(0.09) |  |
| Female | -2.08(0.12)^***^ | -1.85(0.10)^***^ | -0.23(0.06)^***^ |  |
| Age65~70 (Ref. Age60~) | -0.76(0.11)^***^ | -0.42(0.09)^***^ | -0.37(0.05)^***^ |  |
| Age>70 (Ref. Age60~) | -2.61(0.12)^***^ | -1.66(0.09)^***^ | -1.05(0.05)^***^ |  |
| Migrant x Time | 0.16(0.12) | 0.06(0.10) | 0.11(0.06) |  |
| Female x Time | -0.14(0.08) | -0.10(0.10) | -0.05(0.04) |  |
| Migrant x Female | 0.61(0.27)^*^ | 0.43(0.22)^*^ | 0.18(0.12) |  |
| Migrant x Time x Female | -0.15(0.16) | -0.08(0.14) | -0.07(0.08) |  |
| Random effect |  |  |  |  |
| ID | 2.16 | 1.63 | 0.90 |  |
| Time | 0.35 | 0.20 | 0.32 |  |

^*^ *P*<0.05; ^**^ *P*<0.01; ^***^ *P*<0.001;

**Table S2** Longitudinal Association Between the Mental Status and Migrant Status in Female Subjects

| Mixed Effect | β (SE) |  |  |  |  |
| --- | --- | --- | --- | --- | --- |
|  | Model1 | Model2 | Model3 | Model4 | Model5 |
| Fixed effect |  |  |  |  |  |
| constant | 6.10(0.09)^***^ | 5.03(0.12)^***^ | 5.03(0.16)^***^ | 5.43(0.18)^***^ | 5.40(0.19)^***^ |
| Time | -0.59(0.04)^***^ | -0.59(0.04)^***^ | -0.59(0.04)^***^ | -0.59(0.04)^***^ | -0.59(0.04)^***^ |
| Migrant (Ref. Non-migrants) | 0.66(0.12)^***^ | 0.41(0.10)^***^ | 0.41(0.11)^***^ | 0.32(0.10)^**^ | 0.29(0.10)^**^ |
| Age65~70 (Ref. Age60~) | -0.47(0.12)^***^ | -0.47(0.11)^***^ | -0.48(0.11)^***^ | -0.44(0.10)^***^ | -0.43(0.10)^***^ |
| Age>70 (Ref. Age60~) | -1.69(0.13)^***^ | -0.92(0.12)^***^ | -0.88(0.13)^***^ | -0.88(0.12)^***^ | -0.84(0.12)^***^ |
| **SES** |  |  |  |  |  |
| Education level (Ref. illiterate) |  |  |  |  |  |
| Some primary school |  | 1.72(0.12)^***^ | 1.72(0.12)^***^ | 1.70(0.12)^***^ | 1.68(0.12)^***^ |
| Finished primary school |  | 3.03(0.13)^***^ | 3.02(0.13)^***^ | 2.93(0.13)^***^ | 2.89(0.13)^***^ |
| Higher than primary school |  | 3.78(0.23)^***^ | 3.75(0.23)^***^ | 3.61(0.22)^***^ | 3.59(0.22)^***^ |
| Retired (Ref. no) |  | -0.02(0.10) | 0.01(0.10) | 0.01(0.10) | 0.05(0.10) |
| Household annual income (Ref. low) |  |  |  |  |  |
| Medium |  | -0.13(0.10) | -0.07(0.11) | -0.15(0.11) | -0.15(0.11) |
| High |  | 0.28(0.13)^*^ | 0.42(0.14)^**^ | 0.24(0.14)^*^ | 0.23(0.14) |
| **Family connections** |  |  |  |  |  |
| Coupled household |  |  | 0.02(0.11) | 0.03(0.11) | 0.02(0.11) |
| Living with child |  |  | -0.33(0.10)^***^ | -0.27(0.10)^**^ | -0.27(0.10)^**^ |
| Caring for grandchildren |  |  | 0.19(0.09)^*^ | 0.21(0.09)^*^ | 0.22(0.09)^*^ |
| **Social attachment** |  |  |  |  |  |
| Civic participation (Ref.no) |  |  |  | 0.60(0.51) | 0.51(0.51) |
| Leisure activities (Ref.no) |  |  |  | 0.55(0.15)^***^ | 0.52(0.15)^***^ |
| Friendships (Ref.no) |  |  |  | 0.16(0.09) | 0.12(0.09) |
| **Depression** |  |  |  | -0.04(0.01)^***^ | -0.04(0.01)^***^ |
| **Health** |  |  |  |  |  |
| ADLs |  |  |  |  | -0.13(0.04)^***^ |
| Chronic diseases (Ref. Non-disease) |  |  |  |  | -0.06(0.10) |
| **Health behavior** |  |  |  |  |  |
| Smoking (Ref. Non-smoker) |  |  |  |  |  |
| Light/moderate smokers |  |  |  |  | 0.17(0.21) |
| Heavy smokers |  |  |  |  | 0.14(0.33) |
| Alcohol consumption (Ref. Non-drinker) |  |  |  |  |  |
| ≤1 drink per month |  |  |  |  | -0.01(0.22) |
| >1 drink per month |  |  |  |  | -0.02(0.16) |
| Afternoon napping (Ref. Non-napper) |  |  |  |  |  |
| Short napper |  |  |  |  | 0.37(0.17)^*^ |
| Moderate napper |  |  |  |  | 0.25(0.10)^*^ |
| Extend napper |  |  |  |  | -0.09(0.14) |
| Random effect |  |  |  |  |  |
| ID | 1.72 | 1.30 | 1.28 | 1.21 | 1.20 |
| Time | 0.12 | 0.06 | 0.05 | 0.08 | 0.08 |
| AIC | 30385 | 29717 | 29708 | 29645 | 29639 |

Abbreviations: AIC Akaike Information Criterion, SES socioeconomic status, ADL activity of daily living disability

Model 1: Adjusted for age group and time of follow-up; Model 2: Model 1 + socioeconomic status; Model 3: Model 2 + family connections; Model 4: Model3 + social attachment + depression; Model 5: Model 4 + health and health behaviours.

^*^ *P*<0.05; ^**^ *P*<0.01; ^***^ *P*<0.001;

**Table S3** Longitudinal Association Between the Total Cognitive Trajectory and Migrant Status in Male Subjects

| Mixed Effect | β (SE) |  |  |  |  |
| --- | --- | --- | --- | --- | --- |
|  | Model1 | Model2 | Model3 | Model4 | Model5 |
| Fixed effect |  |  |  |  |  |
| constant | 11.36(0.12)^***^ | 8.86(0.19)^***^ | 8.70(0.24)^***^ | 9.08(0.26)^***^ | 8.92(0.28)^***^ |
| Time | -0.81(0.05)^***^ | -0.81(0.05)^***^ | -0.81(0.05)^***^ | -0.81(0.05)^***^ | -0.81(0.05)^***^ |
| Migrant (Ref. Non-migrants) | 0.28(0.17) | 0.22(0.15) | 0.21(0.15) | 0.18(0.15) | 0.16(0.15) |
| Age65~70 (Ref. Age60~) | -0.68(0.16)^***^ | -0.64(0.14)^***^ | -0.65(0.14)^***^ | -0.56(0.14)^***^ | -0.53(0.14)^***^ |
| Age>70 (Ref. Age60~) | -2.52(0.17)^***^ | -1.58(0.16)^***^ | -1.57(0.16)^***^ | -1.57(0.16)^***^ | -1.54(0.16)^***^ |
| **SES** |  |  |  |  |  |
| Education level (Ref. illiterate) |  |  |  |  |  |
| Some primary school |  | 1.80(0.17)^***^ | 1.79(0.17)^***^ | 1.75(0.16)^***^ | 1.74(0.17)^***^ |
| Finished primary school |  | 3.22(0.16)^***^ | 3.19(0.16)^***^ | 3.10(0.16)^***^ | 3.07(0.16)^***^ |
| Higher than primary school |  | 4.36(0.20)^***^ | 4.31(0.20)^***^ | 4.09(0.19)^***^ | 4.04(0.19)^***^ |
| Retired (Ref. no) |  | -0.53(0.15)^***^ | -0.48(0.15)^**^ | -0.46(0.15)^**^ | -0.42(0.15)^**^ |
| Household annual income (Ref. low) |  |  |  |  |  |
| Medium |  | 0.18(0.14) | 0.23(0.14) | 0.23(0.14) | 0.24(0.14) |
| High |  | 0.01(0.18) | 0.16(0.20) | -0.01(0.19) | 0.01(0.19) |
| **Family connections** |  |  |  |  |  |
| Coupled household |  |  | 0.26(0.17) | 0.20(0.17) | 0.20(0.17) |
| Living with child |  |  | -0.31(0.13)^*^ | -0.23(0.13) | -0.24(0.13) |
| Caring for grandchildren |  |  | 0.08(0.13) | 0.07(0.12) | 0.06(0.12) |
| **Social attachment** |  |  |  |  |  |
| Civic participation (Ref.no) |  |  |  | 0.67(0.59) | 0.61(0.59) |
| Leisure activities (Ref.no) |  |  |  | 0.68(0.15)^***^ | 0.63(0.15)^***^ |
| Friendships (Ref.no) |  |  |  | 0.55(0.13)^***^ | 0.51(0.13)^***^ |
| **Depression** |  |  |  | -0.07(0.01)^***^ | -0.07(0.01)^***^ |
| **Health** |  |  |  |  |  |
| ADLs |  |  |  |  | -0.17(0.06)^*^ |
| Chronic diseases (Ref. Non-disease) |  |  |  |  | 0.09(0.13) |
| **Health behavior** |  |  |  |  |  |
| Smoking (Ref. Non-smoker) |  |  |  |  |  |
| Light/moderate smokers |  |  |  |  | 0.12(0.15) |
| Heavy smokers |  |  |  |  | 0.27(0.14) |
| Alcohol consumption (Ref. Non-drinker) |  |  |  |  |  |
| ≤1 drink per month |  |  |  |  | 0.07(0.22) |
| >1 drink per month |  |  |  |  | -0.20(0.12) |
| Afternoon napping (Ref. Non-napper) |  |  |  |  |  |
| Short napper |  |  |  |  | 0.23(0.21) |
| Moderate napper |  |  |  |  | 0.18(0.14) |
| Extend napper |  |  |  |  | 0.07(0.16) |
| Random effect |  |  |  |  |  |
| ID | 2.03 | 1.57 | 1.57 | 1.44 | 1.44 |
| Time | 0.45 | 0.38 | 0.38 | 0.42 | 0.42 |
| AIC | 29626 | 29093 | 29090 | 28988 | 28991 |

Abbreviations: AIC Akaike Information Criterion, SES socioeconomic status, ADL activity of daily living disability

Model 1: Adjusted for age group and time of follow-up; Model 2: Model 1 + socioeconomic status; Model 3: Model 2 + family connections; Model 4: Model3 + social attachment + depression; Model 5: Model 4 + health and health behaviours.

^*^ *P*<0.05; ^**^ *P*<0.01; ^***^ *P*<0.001;

**Table S4** Longitudinal Association Between the Mental Status and Migrant Status in Male Subjects

| Mixed Effect | β (SE) |  |  |  |  |
| --- | --- | --- | --- | --- | --- |
|  | Model1 | Model2 | Model3 | Model4 | Model5 |
| Fixed effect |  |  |  |  |  |
| constant | 7.87(0.09)^***^ | 5.88(0.15)^***^ | 5.78(0.19)^***^ | 6.05(0.20)^***^ | 5.82(0.22)^***^ |
| Time | -0.48(0.04)^***^ | -0.48(0.04)^***^ | -0.48(0.04)^***^ | -0.48(0.04)^***^ | -0.48(0.04)^***^ |
| Migrant (Ref. Non-migrants) | 0.30(0.13)^*^ | 0.23(0.12) | 0.22(0.12) | 0.20(0.11) | 0.19(0.12) |
| Age65~70 (Ref. Age60~) | -0.36(0.12)^**^ | -0.32(0.11)^**^ | -0.32(0.11)^**^ | -0.26(0.11)^*^ | -0.24(0.11)^*^ |
| Age>70 (Ref. Age60~) | -1.61(0.13)^***^ | -0.85(0.12)^***^ | -0.84(0.12)^***^ | -0.84(0.12)^***^ | -0.82(0.12)^***^ |
| **SES** |  |  |  |  |  |
| Education level (Ref. illiterate) |  |  |  |  |  |
| Some primary school |  | 1.53(0.13)^***^ | 1.53(0.13)^***^ | 1.50(0.13)^***^ | 1.48(0.13)^***^ |
| Finished primary school |  | 2.61(0.12)^***^ | 2.59(0.13)^***^ | 2.53(0.12)^***^ | 2.51(0.12)^***^ |
| Higher than primary school |  | 3.22(0.15)^***^ | 3.18(0.15)^***^ | 3.02(0.15)^***^ | 2.98(0.15)^***^ |
| Retired (Ref. no) |  | -0.44(0.12)^***^ | -0.39(0.12)^***^ | -0.38(0.12)^***^ | -0.35(0.12)^**^ |
| Household annual income (Ref. low) |  |  |  |  |  |
| Medium |  | 0.13(0.11) | 0.18(0.11) | 0.19(0.10) | 0.18(0.11) |
| High |  | 0.02(0.14) | 0.18(0.15) | 0.06(0.15) | 0.07(0.15) |
| **Family connections** |  |  |  |  |  |
| Coupled household |  |  | 0.17(0.13) | 0.13(0.13) | 0.12(0.13) |
| Living with child |  |  | -0.30(0.10)^**^ | -0.25(0.10)^*^ | -0.24(0.10)^*^ |
| Caring for grandchildren |  |  | 0.11(0.10) | 0.10(0.09) | 0.09(0.10) |
| **Social attachment** |  |  |  |  |  |
| Civic participation (Ref.no) |  |  |  | 0.52(0.46) | 0.49(0.46) |
| Leisure activities (Ref.no) |  |  |  | 0.58(0.12)^***^ | 0.53(0.12)^***^ |
| Friendships (Ref.no) |  |  |  | 0.31(0.10)^**^ | 0.29(0.10)^**^ |
| **Depression** |  |  |  | -0.05(0.01)^***^ | -0.05(0.01)^***^ |
| **Health** |  |  |  |  |  |
| ADLs |  |  |  |  | -0.12(0.05)^*^ |
| Chronic diseases (Ref. Non-disease) |  |  |  |  | 0.13(0.10) |
| **Health behavior** |  |  |  |  |  |
| Smoking (Ref. Non-smoker) |  |  |  |  |  |
| Light/moderate smokers |  |  |  |  | 0.07(0.11) |
| Heavy smokers |  |  |  |  | 0.20(0.11) |
| Alcohol consumption (Ref. Non-drinker) |  |  |  |  |  |
| ≤1 drink per month |  |  |  |  | -0.04(0.17) |
| >1 drink per month |  |  |  |  | -0.08(0.10) |
| Afternoon napping (Ref. Non-napper) |  |  |  |  |  |
| Short napper |  |  |  |  | 0.21(0.16) |
| Moderate napper |  |  |  |  | 0.22(0.11)^*^ |
| Extend napper |  |  |  |  | 0.15(0.13) |
| Random effect |  |  |  |  |  |
| ID | 1.53 | 1.16 | 1.15 | 1.07 | 1.07 |
| Time | 0.31 | 0.26 | 0.27 | 0.29 | 0.28 |
| AIC | 27275 | 26751 | 26745 | 26661 | 26662 |

Abbreviations: AIC Akaike Information Criterion, SES socioeconomic status, ADL activity of daily living disability

Model 1: Adjusted for age group and time of follow-up; Model 2: Model 1 + socioeconomic status; Model 3: Model 2 + family connections; Model 4: Model3 + social attachment + depression; Model 5: Model 4 + health and health behaviours.

^*^ *P*<0.05; ^**^ *P*<0.01; ^***^ *P*<0.001;
